# Frequency vs. intensity: which should be used as anchors for self-report instruments?

**DOI:** 10.1186/1477-7525-10-107

**Published:** 2012-09-06

**Authors:** Julia Krabbe, Thomas Forkmann

**Affiliations:** 1 Institute of Medical Psychology and Medical Sociology, University Hospital of RWTH Aachen, Pauwelsstraße 19, Aachen, 52074, Germany

## Abstract

**Background:**

The aim of the present study was to investigate the usability of verbal rating scale anchors in patients suffering from a depressive episode and whether differences between frequency or intensity scales could be determined. Frequency and intensity terms were evaluated concerning their interindividual congruency, intraindividual stability across time, and distinguishability of adjacent terms.

**Methods:**

In a longitudinal design, 44 patients (age M=39.1, SD=15.2, 68.2% female) with a depressive disorder filled out several established questionnaires (e.g. BDI or SCL-90) and questionnaires containing frequency and intensity terms which should be indicated by the percentage of time or intensity that is reflected by each term at two different measuring times within one week. Data analysis contained t-tests for paired samples and effect sizes d according to Cohen.

**Results:**

Intensity terms showed weaker intraindividual stability across time as compared to frequency terms. Participants were able to reliably distinguish four frequency and intensity terms at both measuring times. Overall congruency between patients was larger for intensity terms in comparison to frequency terms.

**Conclusions:**

The present results indicate that both frequency and intensity terms can be applied as verbal anchors for clinical self-report scales. However, if longitudinal assessment is intended, our results indicate that frequency terms should be used as they showed slightly greater stability across time. Generally, the present study suggests that no more than four different verbal anchors should be used together in rating scales as especially older patients and those with low lexical experience would not be able to reasonably differentiate more than these.

## Background

The gold standard for diagnosing a mental disorder is a structured diagnostic interview that examines whether the criteria for a respective disorder are fulfilled according to the ICD-10 or DSM-IV [1,2]. In addition, to assess the severity of a mental disorder in case that a diagnosis has already been assigned, numerous standardized instruments are available, mainly self-rating scales (questionnaires) or scales to be filled in by the diagnostician. These questionnaires usually contain a set of statements referring to symptoms associated with the respective disorder together with a rating scale. Respondents are requested to mark on the scale how intensive they experience the symptom or how frequently the symptom occurred in a defined time slot. Most rating scales applied in questionnaires differ with regard to number of categories, point of origin and labeling. Some scales are exclusively labeled by numbers (e.g., from 1 to 5), others are labeled verbally (e.g., from “never” to “always”) or both. Over the last years a considerable amount of research emerged that addressed the effect of several rating scale attributes on the responses of the test taker (for an overview see e.g., [3]).

For example, scales which are labeled by only positive numbers (e.g., from 0 to 6) get different answers than scales with negative and positive numbers (e.g., from -3 to +3). Furthermore, attributes like the number of response options, polarity or whether a rating scale includes “0” as response option have influence on the response behavior of test takers [4-8]. However, despite extensive research on attributes of rating scales and their impact on response behavior, the question how to choose verbal anchors for clinical rating scales has mostly been disregarded and the question whether clinical self-report instruments should rather ask for the frequency or intensity of symptoms has not been investigated empirically so far.

In clinical examination both intensity and frequency of a symptom are important and should be accounted for by the treating therapist. In addition, the relevant diagnostic criteria as described in ICD-10 or DSM-IV usually include both the frequency *and* intensity of symptoms. However, when designing a self-report instrument diagnosticians have to decide which of the two dimensions to consider more important to asses. Both DSM-IV and ICD-10 offer no clear advice which of the two options should be chosen.

A couple of earlier studies from the area of research on medical education addressed this issue from a different perspective. Case [9] asked members of test committees who write questions for medical examinations to indicate the percentage of time or intensity that was reflected by imprecise terms of frequency (e.g. usually) which are commonly used in multiple choice questions. Contrary to the assumption that there is a common definition among medical professionals about the phrases used the results showed virtually no congruence between the professionals’ rating.

Other studies revealed that even terms like “never” or “always” which were expected to be rated as absolute “0%” or “100%” both were indicated with a range up to 20% [10].

In a study about the measurement of fatigue Chang et al. [11] analyzed data from 161 patients (cancer, stroke and HIV) on two 5-point symptom self-report rating scales, one for frequency and one for intensity. Applying Rasch analysis they found a subtle but meaningful advantage for frequency terms providing a fuller coverage of the fatigue continuum. The authors argued that their results could be interpreted as indicating that frequency scales outmatch intensity scales psychometrically.

Taken together, despite its importance for the design of clinical instruments, the question whether frequency or intensity should be used as verbal anchors for self-report rating scales has only rarely been addressed so far. Results suggest that interindividual congruency of mental representations of imprecise terms on frequency or intensity generally appears to be low. However, if respondents are not able to reliably distinguish between terms like “seldom” and “sometimes”, the question arises, whether it is justified to use them in a common scale which is often the case in clinical practice. Thus, it remains an open question which criteria should be applied to decide (1) whether a rating scale should be scaled for intensity or frequency and (2) which terms should be “allowed” to be used together in a common scale.

Therefore, the aim of this study was to search for an empirical basis for criteria to decide whether frequency or intensity scales should be used in clinical self-report instruments. Data from patients with a depressive disorder were acquired for this purpose. We proposed that imprecise terms used as verbal anchors in rating scales should at least adhere to the following basic requirements:

1. interindividual congruency of mental representations of anchor terms

2. intraindividual stability across time of mental representations of anchor terms

3. distinguishability of adjacent terms

These issues were examined for both terms on frequency and intensity. In the light of prior investigations we expected low interindividual congruency and intraindividual stability of mental representations. Practical implications for scale development and refinement as well as suggestions which terms should be allowed in a common rating scale are discussed.

## Methods

### Sample

A total of 44 patients from a German university hospital and several community psychiatry clinics suffering from a depressive disorder according to ICD-10 as leading or secondary diagnosis provided data for this study. A further inclusion criterion was sufficient German language skills. Depression was chosen because it represents one of the most common and thus most important groups of mental disorders [12-14].

When applying statistical power analysis (e.g. using the software G*Power 3.1.3, estimation for point biserial correlations [15,16]) with N = 44 and α = 0.05, the design of the present study has enough power (1-β = 0.96) to detect medium sized effects (d ≥ 0.5). Of course, when considering smaller effects power decreases. However, the present study intended to provide first data on the question whether patients with a depressive disorder show interindividual congruency and intraindividual stability when judging imprecise frequency and intensity terms and to derive suggestions on how many and which such terms may be used in a common scale. Rigorous criteria were considered important for this purpose. Thus, we feel it most important to prevent type-1 errors, i.e. the erroneous rejection of the H_0_, while accepting a slightly heightened type-2 error.

The mean age of the sample was M=39.1 years (SD=15.2) with a range from 17 to 78. Fourteen male and 30 female patients (68.2%) participated in the study. For sample details see Table 1. The study was approved by the local ethic committee and performed according to the Declaration of Helsinki.

**Table 1:** Characteristics of the study sample

		**M**	**SD**
	age	39.1	15.2
	BDI t1*	31.2	11.0
	BDI t2*	26.9	10.7
	SCL GSI (t value) t1*	68.1	10.3
	SCL GSI (t value) t2*	70.7	9.3
		**n**	**percentage (%)**
sex			
	male	14	31.8
	female	30	68.2
nationality			
	German	43	97.7
	Turkish	1	2.3
first language			
	German	42	95.5
	Turkish	1	2.3
	Slovakian	1	2.3
years of education			
	<10 years	1	2.3
	10-13 years	24	54.5
	>13 years	16	36.4
diagnoses	(multiple diagnoses possible)		
	Depressive episode (F32.xx)	25	56.8
	Recurrent depressive disorder (F33.xx)	18	40.9
	Disorder of adult personality (F6x.xx)	9	20.5
	Other anxiety disorder (F41.xx)	4	9.1
	Mental disorder due to psychotic substance use (F1x.xx)	3	6.8
	Bipolar Affective Disorder (currently depressed; F31.3x/ F31.4x)	2	4.5
	Persistent affective disorder (F34.xx)	2	4.5
	Adjustment disorder (F43.2x)	1	2.3
	Schizophrenia (F2x.xx)	1	2.3
	Agoraphobia (F40.0x)	1	2.3

*test interval t2-t1 = one week.

## Material

### Beck Depression Inventory (BDI)

The BDI [17] contains 21 items. Each item consists of four self-referring statements (e.g. “I am sad”). Item scores range from 0 to 3 and participants are supposed to choose one or more statements per item that represents best their mental state during the last week. A total score >10 indicates mild to moderate depression and a total score >18 moderate to severe depression.

### The Symptom Checklist-90-revised (SCL-90)

The SCL-90 [18] contains 90 items that are Likert-scaled, referring to the previous week, with a range from 0 (“not at all”) to 4 (“very much”). The instrument provides information on overall psychological distress. Furthermore, the 90 items of the inventory constitute three composite scores and nine symptom scales (Somatisation, Obsessive-Compulsive, Interpersonal Sensitivity, Depression, Anxiety, Hostility, Phobic Anxiety, Paranoid Ideation, Psychoticism) allowing the calculation of psychopathological profiles. The three composite scores reflect the complete answer pattern of the respondent: the “global severity index” (GSI) measures the overall mental symptom burden, the “positive symptom distress index” (PSDI) measures symptom intensity, and the “positive symptom total” (PST) reflects the total number of the respondent’s symptoms. The raw scale and composite scores are transformed to standardized T-scores with a mean of 50 and a standard deviation (SD) of 10. T-scores >60 reflect heightened mental burden. For our analyses we only used the GSI as indicator of general symptom burden.

### German vocabulary test (“Wortschatztest”, WST)

The WST [19] contains 40 items. Each item consists of six words of which only one is a real word which can be found in a German dictionary. The participants are supposed to choose and highlight the real word. The number of correctly chosen real words creates the raw score which can be linearly transformed into various standardized scores. In this study, a transformation into z-scores (M=0, SD=1) was performed.

### Questionnaires about frequency and intensity

In a first step of the construction of material for the measurement of mental representations of terms on frequency and intensity a pool of terms commonly used in rating scales of self-report instruments was compiled. For this purpose, established questionnaires in German (34 using intensity, 35 using frequency scales; a complete list of all screened questionnaires is available on request from the principal author) were screened resulting in a pool of fifteen terms on intensity (e.g., “very much”) and fourteen terms on frequency (e.g., “sometimes”). Only those terms were included which showed an appearance in at least 10% of the screened questionnaires to create a nearly equal number of phrases for both frequency and intensity. The threshold of 10% was also chosen to prevent that random phrases which appear only in single questionnaires would be assessed in the study. Patients were asked to indicate the percentage of time or intensity that is reflected by each term.

### Further materials

All patients completed a demographic data sheet. Clinical data were taken from a data sheet which was filled out by the treating therapist.

#### Procedures

Questionnaire fulfilment was explained and supervised either by the principal author or the treating psychotherapist. All patients took part voluntarily without payment and signed an informed consent prior to testing. The therapists received a reward of 10€ for recruiting, administering and returning the questionnaire packages.

Patients were required to fill in the BDI, SCL-90 and the questionnaires about frequency and intensity twice within an interval of one week (t_1_ and t_2_). WST and demographic data sheet were administered only once at t_1_.

### Data analysis

#### Interindividual congruency of mental representations of anchor terms

Congruency of mental representations of frequency terms was compared to intensity terms by means of t-tests for paired samples and effect sizes *d* between the mean standard deviation of frequency terms (SD_freq_) and intensity terms (SD_int_) and their confidence intervals (95%). If the confidence interval for *d* includes zero, the effect can be regarded as statistically nonsignificant. In order to reduce sampling error effect sizes have been corrected using a factor provided by Hedges and Olkin [20]. Following Cohen [21] effect sizes .20<d≤.50 were interpreted as small, .50<d≤.80 as medium, and d>.80 as large.

Furthermore, we investigated whether interindividual congruency differed in dependence on patients’ age, gender, vocabulary, depression (BDI) or overall mental symptom burden (GSI). For this purpose, the sample was divided by median split on the respective variable (e.g., age) and pair wise comparisons using t-tests and effect sizes *d* were conducted.

#### Intraindividual stability across time of mental representations of anchor terms

For the determination of the intraindividual stability of mental representations of anchor terms patients’ ratings on frequency and intensity terms on t_1_ and t_2_ were compared using t-tests for paired samples and effect sizes *d* according to Cohen. Significant t-tests and effect sizes *d* >.20 that do not include “0” were considered as signs of intraindividual instability of mental representations. In order to identify the phrases which show a strong intraindividual stability it was considered important to apply rather strict standards. So even the smallest effect plus significance in the students' t-test were considered as an indication for instability.

#### Distinguishability of adjacent terms

To assess patients’ ability to distinguish adjacent terms analysis of effect sizes and their confidence intervals were calculated according to the method used to assess interindividual congruency. Following Cohen effect sizes .20<d≤.50 were interpreted as small, .50<d≤.80 as medium, and d>.80 as large. The number of distinguishable adjacent terms was determined for small, medium and large effects between terms separately.

## Results

### Interindividual congruency of mental representations

Overall interindividual congruency was poor for both frequency (M_SDt1_= 20.06; M_SDt2_= 19.90) and intensity terms (M_SDt1_= 18.31; M_SDt2_= 15.68) but larger for intensity terms in comparison to frequency terms. Its difference was significant only for t_2_ (t_1_: *d=.26*, CI [-.16 - .68], t=.30 (p=.77); t_2_: *d=.78*, CI [.34 - 1.21], t=2.57 (p=.02)). The congruency of both frequency and intensity terms was influenced by age and gender. Younger (age↓) and male patients showed a larger congruency than older (age↑) and female patients (Tables 2 and 3).

**Table 2:** Interindividual congruency of mental representations of frequency terms

	**t**_**1**_				**t**_**2**_			
	**M**_**1**_^**#**^**(SD**_**1**_**)**	**M**_**2**_^**#**^**(SD**_**2**_**)**	**t(p)**	**d(CI)**	**M**_**1**_^**#**^**(SD**_**1**_**)**	**M**_**2**_^**#**^**(SD**_**2**_**)**	**t(p)**	**d(CI)**
WST	18.61(5.54)	19.70(9.23)	-.37(.72)	-.14(-.73-.45)	21.67(6.49)	17.37(7.90)	1.52(.14)	.59(-.02-1.18)
age	14.58(5.07)	23.53(7.46)	−3.58(.00)^*^	−1.40(-2.03--.71)	16.81(5.34)	21.68(6.97)	−2.00(.06)	-.77(-1.39--.15)°
sex	9.44(4.68)	22.62(6.57)	−5.82(.00)^*^	−2.16(-2.9--1.35)°	13.33(7.24)	21.92(6.57)	−3.17(.00)^*^	−1.27(-1.93--.56)°
BDIt1	19.46(7.35)	19.55(4.95)	-.03(.97)	-.01(-.61-.58)	18.13(7.01)	19.51(6.55)	-.52(.61)	-.21(-.80-.40)
BDIt2	22.26(7.80)	16.42(5.77)	2.17(.04)^*^	.85(.20-.48)°	20.92(6.85)	17.71(6.46)	1.23(.23)	.48(-.15-1.09)
SCLt1	20.08(7.80)	15.75(6.63)	1.53(.14)	.60(-.90-1.26)	17.33(8.50)	19.63(6.89)	-.76(.46)	-.30(-.96-.37)
SCLt2	18.02(9.26)	19.97(4.01)	-.13(.74)	-.14(-.79-.52)	14.67(8.93)	21.10(7.31)	−2.01(.06)	-.79(-1.45--.19)°

^#^ M1 refers to: WST↓, age↓, male, BDI t1 ↓, BDI t2 ↓, SCL t1 ↓ and SCL t2 ↓; M2 refers to: WST↑, age↑, female, BDI t1↑, BDI t2↑, SCL t1↑ and SCL t2↑.

^*^ p<.05.

° If the effect size d is larger than .20 and the conf idence interval for d does not include zero, the effect can be regarded as statistically significant.

**Table 3:** Interindividual congruency of mental representations of intensity terms

	**t**_**1**_				**t**_**2**_			
	**M**_**1**_^**#**^**(SD**_**1**_**)**	**M**_**2**_^**#**^**(SD**_**2**_**)**	**t(p)**	**d(CI)**	**M**_**1**_^**#**^**(SD**_**1**_**)**	**M**_**2**_^**#**^**(SD**_**2**_**)**	**t(p)**	**d(CI)**
WST	17.94(7.23)	17.63(10.33)	.09(.93)	.03(.-56-.62)	21.67(6.49)	13.90(5.67)	1.68(.11)	1.28(.60-1.91)°
age	11.86(4.55)	22.29(10.70)	-3.47(.00)^*^	-1.18(-1.89--.43)°	11.63(5.26)	18.08(6.67)	-2.94(.01)^*^	1.05(-1.73--.31)°
sex	8.13(5.47)	20.60(9.44)	-4.43(.00)^*^	-1.48(-2.16--.75)°	7.90(5.04)	17.69(4.87)	-5.41(.00)^*^	-1.99(-2.72--1.18)°
BDIt1	17.39(7.88)	18.04(8.80)	-.22(.83)	-.08(-.68-.52)	13.35(5.67)	14.82(5.40)	-.73(.47)	-.27(-.87-.35)
BDIt2	19.44(8.67)	16.41(8.58)	.96(.34)	.35(-.27-.96)	13.64(5.73)	14.77(6.01)	-.53(.60)	-.19(-.81-.43)
SCLt1	19.44(8.67)	16.41(8.58)	.96(.34)	.35(-.27-.96)	17.69(5.00)	15.45(6.74)	.74(.47)	.38(-.31-1.05)
SCLt2	19.41(10.12)	18.98(6.94)	.21(.84)	.02(-.64-.67)	16.15(5.34)	12.73(5.59)	.82(.42)	.63(-.60-1.28)

^#^ M1 refers to: WST↓, age↓, male, BDI t1 ↓, BDI t2 ↓, SCL t1 ↓ and SCL t2 ↓; M2 refers to: WST↑, age↑, female, BDI t1↑, BDI t2↑, SCL t1↑ and SCL t2↑.

* p<.05.

° If the effect size d is larger than .20 and the confidence interval for d does not include zero, the effect can be regarded as statistically significant.

### Intraindividual stability of mental representations

**Figure 1:**
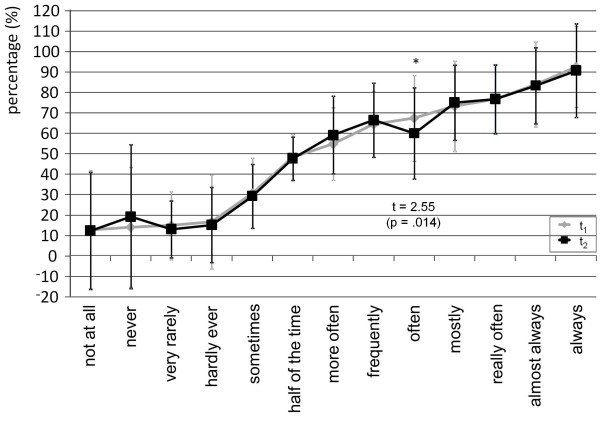
Intraindividual stability of mental representations of frequency terms.

**Figure 2:**
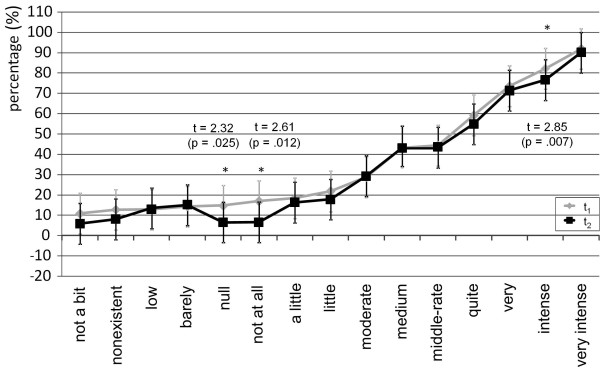
Intraindividual stability of mental representations of intensity terms.

On single item level most terms showed sufficient intraindividual stability considering that both t-tests and effect sizes showed no statistical significances. According to the performed t-tests, intensity terms showed intraindividual instability for three terms (no, not all, intense) while there was only one frequency term (often) which was not intraindividual stable (Figures 1 and 2).

When aggregating data, i.e. when averaging the standard deviations of all items, intraindividual stability across time was evident for both frequency terms and intensity terms. However, patients reporting higher levels of depression as indicated by a high BDI sum score at t_1_ (BDI t_1_ ↑) and patients reporting higher levels of general mental distress as indicated by a high SCL GSI score at t_2_ (SCL t_2_ ↑) judged intensity terms more heterogeneously at t_1_ than at t_2_ according to effect size (t_BDI_↑=2.474 (p=.027) and ES_SCL_↑=.992 (CI .299 - 1.644) respectively) (Tables 4 and 5).

**Table 4:** Distinguishability of adjacent terms and intraindividual stability of frequency terms

	**distinguishable term**						**intraindividual stability**			
	**n**_**t1**_			**n**_**t2**_						
	**20<d≤.50**	**.50<d≤.80**	**d>.80**	**.20<d≤.50**	**.50<d≤.80**	**d>.80**	**M**_**1**_**(SD**_**1**_**)**	**M**_**2**_**(SD**_**2**_**)**	**t(p)**	**d(CI)**
all	7	6	5	6	6	5	20.06(5.10)	19.90(6.34)	.20(.85)	.03(-.39-.45)
WST↓	6	6	5	4	4	4	18.61(5.54)	21.67(6.49)	−1.43(.18)	-.51(-1.11-.12)
WST↑	7	7	7	6	6	6	19.70(9.23)	17.37(7.90)	1.50(.16)	.27(-.31-.85)
age↓	7	7	6	6	6	5	14.58(5.07)	16.81(5.34)	−1.83(.09)	-.43(-1.03-.19)
age↑	5	5	4	4	4	4	23.53(7.46)	21.68(6.97)	.71(.49)	.26(-.34-.85)
male	9	9	9	6	6	6	9.44(4.86)	13.33(7.24)	−1.39(.86)	-.63(-1.37-.15)
female	6	6	5	6	6	4	22.62(6.57)	21.92(6.57)	.81(.43)	.11(-.40-.61)
BDIt1↓	6	6	5	6	6	5	19.46(7.35)	18.13(7.01)	.81(.43)	.19(-.42-.79)
BDIt1↑	6	6	5	5	5	5	19.55(4.95)	19.51(6.55)	.02(.98)	.01(-.59-.60)
BDIt2↓	4	4	4	6	6	5	22.26(7.80)	20.92(6.85)	.73(.48)	.18(-.14-.80)
BDIt2↑	6	6	6	6	6	5	16.42(5.77)	17.71(6.46)	-.79(.45)	-.21(-.81-.40)
SCLt1↓	6	6	5	6	6	6	20.08(7.80)	17.33(8.50)	1.92(.08)	.34(-.35-1.01)
SCLt1↑	7	7	7	5	5	4	15.75(6.63)	19.63(6.89)	−1.40(.19)	-.58(-1.23-.10)
SCLt2↓	5	5	5	7	7	6	18.02(9.26)	14.67(8.93)	2.09(.06)	.37(-.32-1.04)
SCLt2↑	6	6	5	5	5	4	18.97(4.02)	21.10(7.31)	−1.04(.32)	-.36(-1.0-.29)

° If the effect size d is larger than .20 and the confidence interval for d does not include zero, the effect can be regarded as statistically significant.

* p< .05.

**Table 5:** Distinguishability of adjacent terms and intraindividual stability of intensity terms

	**distinguishable term**						**intraindividual stability**			
	**n**_**t1**_			**n**_**t2**_						
	**20<d≤.50**	**.50<d≤.80**	**d>.80**	**.20<d≤.80**	**.50<d≤.80**	**d>.80**	**M**_**1**_**(SD**_**1**_**)**	**M**_**2**_**(SD**_**2**_**)**	**t(p)**	**d(CI)**
all	8	8	5	7	7	5	15.67(4.24)	18.31(7.99)	1.80(.09)	.41(-.02-.83)
WST↓	5	5	5	7	7	5	16.93(4.12)	17.94(7.23)	.76(.45)	.17(-.44-.77)
WST↑	6	6	6	7	7	6	13.90(5.67)	17.63(10.33)	1.81(.09)	.45(-.15-1.03)
age↓	8	8	7	6	6	6	11.63(5.26)	11.86(4.55)	.16(.87)	.05(-.56-1.07)
age↑	5	5	5	6	6	5	18.08(6.67)	22.29(10.71)	1.36(.20)	.47(-.15-1.07)
male	8	8	8	7	7	7	7.90(5.04)	8.13(5.47)	.18(.19)	.04(-.17-.80)
female	7	7	5	7	7	5	17.69(4.87)	20.60(9.44)	1.78(.10)	.39(-.13-.89)
BDIt1↓	5	5	5	7	7	5	13.35(5.67)	17.39(7.88)	1.32(.21)	.59(-.41-1.19)
BDIt1↑	7	7	4	5	5	4	14.82(5.40)	18.04(8.80)	2.47(.03)*	.44(-.17-1.04)
BDIt2↓	5	5	5	6	6	5	20.92(6.85)	22.26(7.80)	1.79(.10)	.18(-.44-.80)
BDIt2↑	8	8	5	6	6	5	14.77(6.01)	16.41(8.58)	.58(.57)	.22(-.40-.83)
SCLt1↓	5	5	5	6	6	6	17.69(5.00)	19.12(8.09)	1.05(.31)	.21(-.47-.88)
SCLt1↑	6	6	5	5	5	5	15.45(6.74)	17.98(8.48)	1.59(.14)	.33(-.35-.99)
SCLt2↓	5	5	5	6	6	5	16.15(5.34)	19.14(10.12)	1.80(.09)	.37(-.32-.104)
SCLt2↑	6	6	5	7	7	6	12.73(5.59)	18.98(6.94)	1.74(.10)	.99(.30-1.64)°

° If the effect size d is larger than .20 and the confidence interval for d does not include zero, the effect can be regarded as statistically significant.

* p< .05.

The data on single item level for every split group can be found in the Additional files 1 and 2.

### Distinguishability of adjacent terms

The distinguishability of adjacent terms was tested for the whole group and for each median split subgroup. The number of distinguishable adjacent terms was determined for small, medium and large effects between terms separately. Details on this analysis can be found in Tables 4 and 5. In the following, main results will be summarized.

In the whole sample the patients were able to distinguish five to seven frequency terms depending on the effect size criterion applied and five to eight intensity terms at both time points.

*WST:* The patients with lower vocabulary skills (WST ↓) were able to differentiate four to six frequency terms and five to seven intensity terms while patients with a higher vocabulary (WST↑) were able to distinguish six to seven terms for both frequency and intensity terms at both time points.

*Age:* Five to seven frequency terms and six to eight intensity terms at both measuring times could be distinguished by the younger patients (age↓), for the older patients (age↑) fewer terms (four to five frequency terms and five to six intensity terms) could be considered.

*Gender:* The distinguishability of terms of the male patients was given for nine (t_1_) respectively six (t_2_) frequency and eight (t_1_) respectively seven (t_2_) intensity terms. Female patients could only distinguish four to six frequency and five to seven intensity terms.

*BDI:* In relation to the BDI sum there was no clear difference between the split groups. The terms that could be distinguished differed about one term more for intensity than frequency.

*SCL*: The SCL split groups showed no clear difference in relation to the distinguishability at the two time points, as well as to frequency and intensity terms.

## Discussion

The aim of the present study was to investigate the usability of verbal rating scale anchors in patients suffering from a depressive episode and to search for an empirical basis for criteria to decide whether frequency or intensity scales should be used in clinical self-report instruments. Three criteria were applied to compare the appropriateness of using frequency as compared to intensity terms in self-report rating scales: (1) interindividual congruency of mental representations of terms, (2) intraindividual stability across time of mental representations of terms, and (3) distinguishability of adjacent terms.

All in all, the reported results do not give a clear picture on whether frequency or intensity terms should be preferred as verbal anchors in rating scales. Intensity terms showed a larger congruency than frequency terms, but however both congruencies were rather low. The congruency of both frequency and intensity terms was influenced by age and gender. Male patients and younger patients seem to show a higher agreement in comparison to female and older (≥38 years) patients in regard to this criterion. However, the majority of patients with a depression are female and older than 50 years [13] and this group showed particularly low congruency when evaluating imprecise terms in the present study. This should be kept in mind when using self-report instruments and should encourage clinicians not to rely on questionnaires alone but rather apply structured diagnostic interviews for diagnostic purposes more frequently, especially in this patient population. When developing new questionnaires diagnosticians might want to consider applying those terms that showed reasonable congruency for older and female patients, too.

In comparison to frequency terms intensity terms showed a higher number of intraindividual instable terms (three vs. one) and instability was additionally influenced by two of the examined additional variables (depression, overall mental symptom burden). This can be interpreted as indicating, that participants differed more in their mental representations of intensity terms than of frequency terms and that severity of mental symptoms (especially depression) inflated these differences more clearly for intensity than for frequency terms. So, concerning interindividual stability frequency terms appear to be slightly superior to intensity terms.

Concerning the distinguishability of adjacent terms no clear general advantage for neither frequency nor intensity terms could be determined. Assessing the distinguishability for the *overall group* there seemed to be a slight advantage for intensity terms. Considering only the strictest criterion of d>.80 there was no difference: for both frequency and intensity terms five terms could be distinguished. The distinguishability of both frequency and intensity terms seemed to be influenced by age and gender as well as lexical experience. Again, particularly older and female patients and those with low lexical experience showed poorer ability to discriminate between adjacent terms. Only four terms could be distinguished by these subgroups. So, these results suggest that rating scales in newly developed questionnaires that are intended to be applied in patients suffering from a depressive disorder should be limited to not more than four different verbal anchors.

The results of this study are generally consistent to the findings of prior research. Chang et al. [11] examined the evaluation of frequency terms of chronic fatigue patients using Rasch analysis and found a subtle but meaningful advantage for frequency terms providing a fuller coverage of the fatigue continuum. They argued that their results could be interpreted as indicating that frequency scales outmatch intensity scales psychometrically. We also found a slight advantage for frequency terms in regard to intraindividual stability, adding further evidence to the assumption that frequency scales might be easier to use for patients than intensity scales. However, it has to be kept in mind, that we found virtually no differences with regard to distinguishability.

Clinically, there is a difference between depression as an affective disorder and its symptoms and fatigue as a symptom accompanying certain medical diseases such as HIV or Cancer as assessed by Chang et al. [11]. However, measuring fatigue in the course of chronic illness Chang and colleagues [11] used similar items for measuring depressive symptoms, e.g. questions about trouble starting activities, tiredness, fatigue or ability to do usual activities. Thus, in this respect our results may be deemed comparable to those reported by Chang et al. [11]. Nevertheless, additional research is needed to investigate whether results generalize across different mental disorders and equally apply to large-scale population based samples.

Case's findings [9] from the area of research on medical education showed that there is only poor congruence between medical professionals’ ratings about the frequency terms used in medical multiple choice examinations. The present study suggests that this result also applies for clinical applications in terms that there is also only poor congruency between patients with a depressive disorder about frequency and intensity terms.

In a study by Holsgrove and Elzubeir [10] terms like “never” or “always” which were expected to be rated as absolute “0%” or “100%” were indicated with a range up to 20%, similar to the patients' assessments in the present study, "never" was rated with a mean of 16% and "always" with a mean of 90%.

Some potential limitations should be considered when interpreting the results reported in this study. The sample size (n=44) was not large so that the stability of the applied statistics might be regarded as limited. However, we applied a longitudinal design which improves statistical power for all comparisons concerning intraindividual stability and power analysis indicated that the design of the present study was not underpowered to detect the effects we were interested in. To ensure homogeneity of the recruited patient sample and because it represents one of the most common and thus important groups of mental disorders [12-14] only patients with a depression as leading or secondary diagnosis were included and their indications have not been compared and adjusted to the results of a control group. Therefore, the degree to which the present results can be generalized to patients with other mental disorders or patients without mental disorders might be limited and additional research is needed to investigate whether our results apply to other patients who are frequently subject to self-report assessments (e.g., patients with anxiety disorders) or large-scale population based investigations in which questionnaires might be applied as screeners for mental disorders.

While it can be assumed that the percentage scaling used in the present study is intuitively understandable when judging intensity terms this might not apply to frequency terms in the same extent. So it could be possible that patients had more difficulties indicating intensity terms by ranking them in their personal range of understanding. However, since results do not indicate that frequency or intensity terms can be deemed superior to the other regarding *all* three criteria evaluated in the present study but rather show a mixed picture there is no evidence that this potential bias might have affected the results systematically.

There was no supervision of the patients while they were filling out the material so it can not be ruled out that some patients might have had problems grasping all instructions. However, the treating therapists who handed over the questionnaire package were explicitly advised to explain the assignment in detail and according to therapists’ feedback all patients reported to understand all instructions.

The study was carried out in German and the used terms were all extracted from commonly used self-report instruments that were developed or translated in German. Therefore the terms in the present study might not all exactly correspond between English and German, so the reader should have in mind that some terms which seem synonymic in English are not in German. Despite this limitation it has to be noted that in the study by Case [9] individuals without mental disorders showed poor congruency similar to what we found in our data although Case’s study was carried out in English. So given the limited previous research on this issue it can tentatively be assumed that those findings could be reproduced in different languages.

To sum up, the reported results suggest that frequency terms seem to have a slight advantage over intensity terms in regard to higher intraindividual stability of mental representations while both groups of terms exhibited low interindividual congruency. Furthermore, from a psychometric perspective, patients differed in their ability to distinguish between different frequency terms and different intensity terms, respectively. If it is intended, that a given rating scale could be applied to *all* patients with a depressive disorder independently of further patient characteristics (i.e., including older patients and those with low lexical experience) then no more than four different verbal anchors should be used.

## Conclusions

The present results do not support a clear recommendation on whether to choose frequency or intensity terms as verbal anchors of self-report rating scales in clinical applications. There is some preliminary evidence that frequency terms might have a slight advantage over intensity terms with regard to intraindividual stability across time so it might be advisable to use frequency terms when designing a self-report instrument that is intended to be applied in longitudinal assessments. Moreover, the present study suggests that no more than four different verbal anchors should be used together in rating scales as patients with a depressive disorder would not be able to reasonably differentiate more than these four. Generally, the results indicate that mental representations of imprecise terms on frequency or intensity can differ depending on patient characteristics (e.g., age, gender, mental symptom burden, lexical experience). Scale developers should account for this issue and carefully deliberate about which and how many terms to be used in a rating scale. Further research should investigate to what extent these results generalize to patients with other mental disorders.

## Competing interests

The authors declare that they have no competing interests.

## Authors' contributions

JK contributed to conception and design of the study, coordinated the data acquisition, conducted the statistical analysis and wrote the manuscript. TF has been involved in drafting and revising the manuscript, and coordinated the study and data acquisition. All authors read and approved the final manuscript.

## Supplementary Material

Additional file 1 Data of frequency terms on single item level for all split groups. Click here for file

Additional file 2 Data of intensity terms on single item level for all split groups. Click here for file
